# Managing potential adverse events during treatment with enfortumab vedotin + pembrolizumab in patients with advanced urothelial cancer

**DOI:** 10.3389/fonc.2024.1326715

**Published:** 2024-04-22

**Authors:** Blaine Brower, Asia McCoy, Hiba Ahmad, Cheryl Eitman, I. Alex Bowman, Jennifer Rembisz, Matthew I. Milowsky

**Affiliations:** ^1^ Division of Oncology, Department of Medicine, University of North Carolina at Chapel Hill, Chapel Hill, NC, United States; ^2^ Genitourinary Medical Oncology, Memorial Sloan Kettering Cancer Center, New York, NY, United States; ^3^ Anschutz Cancer Center – GU Oncology Department, University of Colorado, Aurora, CO, United States; ^4^ University Hospitals Seidman Cancer Center, Case Western Reserve University, Cleveland, OH, United States; ^5^ Genitourinary Oncology, Banner MD Anderson Cancer Center, Gilbert, AZ, United States; ^6^ Department of Genitourinary Oncology, Moffitt Cancer Center, Tampa, FL, United States; ^7^ Lineberger Comprehensive Cancer Center, University of North Carolina at Chapel Hill, Chapel Hill, NC, United States

**Keywords:** enfortumab vedotin, pembrolizumab, urothelial cancer, anticancer therapy, adverse events, immune-related adverse events, irAE

## Abstract

Cisplatin-based chemotherapy has been the standard of care for patients with locally advanced or metastatic urothelial cancer (la/mUC). Enfortumab vedotin, an antibody-drug conjugate directed to Nectin-4, and pembrolizumab, an immune checkpoint inhibitor, are two therapies that have individually provided a survival benefit in patients with la/mUC. The combination regimen of enfortumab vedotin plus pembrolizumab was evaluated in EV-302 (KEYNOTE-A39; NCT0422385), a phase 3 study that showed statistically significant and clinically meaningful improvement in overall survival, progression-free survival, and a key secondary endpoint of overall response rate versus chemotherapy. Based on these results and those from the EV-103 (KEYNOTE-869; NCT03288545) Dose Escalation cohort, Cohort A, and Cohort K, enfortumab vedotin plus pembrolizumab was granted approval from the US Food and Drug Administration for the treatment of adults with la/mUC. While guidelines and recommendations for the management of adverse events (AEs) have been developed for immune checkpoint inhibitor monotherapy and enfortumab vedotin monotherapy, additional guidance is needed for managing AEs that occur with enfortumab vedotin plus pembrolizumab. As monotherapies, enfortumab vedotin and pembrolizumab are both associated with some of the AEs observed with the combination, such as skin reactions, pneumonitis, and diarrhea, which may confound the attribution of the AE to a specific agent and thereby complicate clinical management. In this manuscript, we aim to provide recommendations for best practice for patient care and the management of AEs of clinical interest for patients with la/mUC receiving enfortumab vedotin plus pembrolizumab, including skin reactions, peripheral neuropathy, hyperglycemia, and pneumonitis. These recommendations were developed based on published guidelines, expert opinions, and the clinical experience of the authors, which include oncologist, advanced practice provider, nursing, and pharmacy perspectives. In addition, guidance on patient education and communication is provided. With vigilant monitoring, early detection, and prompt intervention of treatment-emergent AEs based on recommended approaches described herein, it is the authors’ experience that most AEs can be managed with supportive therapy and dose modification/interruptions, allowing patients to continue treatment.

## 1 Introduction

The incidence of urothelial cancer is increasing worldwide and patients with locally advanced or metastatic urothelial cancer (la/mUC) have a particularly poor prognosis (1–4) and a low 5-year survival rate (5). Although cisplatin-based chemotherapy has been the standard treatment of la/mUC for decades (6, 7), most patients will progress within 7 months, and long-term survival remains poor (median overall survival [OS] of ~16–19 months) (8–10); moreover, up to half of patients are ineligible for cisplatin (9, 10). For cisplatin-ineligible patients, a carboplatin-based regimen may be used; however, this regimen is associated with a lower median OS (~11–13 months) (10–13) and tolerability remains poor. Although avelumab maintenance has been shown to improve OS in patients who had not progressed following first-line platinum-containing chemotherapy (7, 14), emerging real-world evidence suggests that approximately half of patients treated with platinum-based chemotherapy are not eligible to receive avelumab maintenance, primarily due to disease progression or death (15).

For those patients ineligible for any platinum-based therapy, immune checkpoint inhibitor (ICI) monotherapy represents a viable treatment option (7); however, objective responses only occur in approximately 20–30% of patients unselected for programmed cell death-ligand 1 (PD-L1) expression (16, 17).

Enfortumab vedotin (EV) and pembrolizumab (Pembro) are two therapies that have individually shown OS benefit in patients with la/mUC (18–21). EV is an antibody-drug conjugate (ADC) directed to Nectin-4 and consists of a monoclonal antibody attached to the microtubule-disrupting agent monomethyl auristatin E (MMAE) via a protease cleavable linker (22), while Pembro is an ICI targeting programmed cell death protein 1 (PD-1) (23). Preclinical data have shown that EV induces immunogenic cell death, and that the combination of EV and a PD-1 inhibitor may enhance antitumor activity compared to each agent alone due to their distinct and complementary engagement of the immune system (24–26).

The safety and efficacy of EV in combination with Pembro (EV + Pembro) has been studied in a phase 1b/2, open-label, multi-cohort study (EV-103/KEYNOTE-869; NCT03288545) (25, 26) and an open-label, randomized phase 3 study (EV-302/KEYNOTE-A39; NCT04223856) (10). In both studies, patients received EV 1.25 mg/kg as a 30-minute intravenous (IV) infusion on days 1 and 8 of a 21-day cycle, followed by an IV infusion of Pembro 200 mg on day 1 of a 21-day cycle. In EV-103, a total of 121 cisplatin-ineligible patients were enrolled across three cohorts: Dose Escalation Cohort (n = 5), Cohort A (n = 40), and Cohort K (n = 76); the primary objectives were safety and overall response rate (ORR) (25, 26). EV + Pembro demonstrated an ORR of 68% (95% confidence interval [CI]: 58.7–76.0) with a generally manageable safety profile (22). In EV-302, a total of 886 patients with previously untreated la/mUC were randomized, with dual primary endpoints of OS and progression-free survival (PFS). EV + Pembro demonstrated statistically significant improvements in OS and PFS versus platinum-based chemotherapy in patients with previously untreated la/mUC, nearly doubling both median OS (31.5 months versus 16.1 months; P<0.00001) and median PFS (12.5 months versus 6.3 months; P<0.00001). EV + Pembro also significantly increased ORR over platinum-based chemotherapy (67.7% [29.1% complete response] versus 44.4% [12.5% complete response]; P<0.00001). Results were consistent regardless of cisplatin-eligibility, the presence of liver metastases, and PD-L1 expression (10), and the safety profile was consistent with that seen in EV-103 (10, 25–27). Based on the results from EV-302, in December 2023 the US Food and Drug Administration approved EV + Pembro for the treatment of adults with la/mUC (28) and was subsequently, added to the National Comprehensive Cancer Network as a preferred treatment option regardless of cisplatin eligibility (7).

Although recommendations have been developed for the management of treatment-emergent AEs from ICI monotherapy (29–32) and EV monotherapy (33, 34), limited guidance is available for managing AEs that occur with EV + Pembro. In particular, EV and Pembro both contribute to some of the AEs observed, which may confound the timely attribution and clinical management of the AE.

Here, we aim to provide best practice for the oncology team on patient care and management of AEs for patients with la/mUC receiving EV + Pembro with the intent of improving the patient experience and outcomes with this novel combination. These recommendations are based on published guidelines, expert opinion, and clinical experience from a diverse group of healthcare providers (HCPs).

## 2 Safety of EV + Pembro in EV-302

In EV-302, the median duration of exposure was 7 months (range: 0.3 to 31.9 months; median of 9 cycles) for EV and 8.5 months (range: 0.3 to 28.5 months; median of 11 cycles) for Pembro. Patients received EV + Pembro (or at least one agent in the case one was discontinued) for a median of 9.4 months (range, 0.3 to 31.9 months; median of 12 cycles) (10, 22, 23). The most common treatment-emergent AEs for EV + Pembro included rash (68%), peripheral neuropathy (67%), and fatigue (51%) (**Table 1**) (22). The most common grade 3–4 treatment-emergent AEs were rash (15%), peripheral neuropathy (8%), and fatigue (6%) (22). Fatal AEs occurred in 3.9% of patients; 0.9% were considered related to treatment with EV + Pembro (one patient each with multiple organ dysfunction syndrome, immune-mediated lung disease, diarrhea, and asthenia) (10, 22). Thirty-five percent of patients experienced an AE that led to permanent discontinuation of EV, while 73% and 42% of patients experienced an AE that led to dose interruption and dose reduction of EV, respectively (22). Twenty-seven percent of patients experienced an AE that led to permanent discontinuation of Pembro and 61% of patients experienced an AE that led to dose interruption of Pembro (23). Dose modifications due to AEs of clinical interest that occurred in patients who received EV + Pembro in EV-302 are shown in **Supplementary Table 1**.

**Table 1:** Adverse reactions and selected laboratory abnormalities ≥20% (all grades) in patients treated with EV + Pembro in EV-302/KEYNOTE-A39 (22, 23).

Adverse reactions	All grades, %	Grade 3–4, %
Skin and subcutaneous tissue disorders		
Rash^*^	68	15
Pruritus	41	1.1
Alopecia	35	0.5
Nervous system disorders		
Peripheral neuropathy^*^	67	8
Dysgeusia	21	0
Metabolism and nutrition disorders		
Decreased appetite	33	1.8
General disorders and administration site conditions		
Fatigue^*^	51	6
Gastrointestinal disorders		
Diarrhea	38	4.5
Nausea	26	1.6
Constipation	26	0
Investigations		
Decreased weight	33	3.6
Eye disorders		
Dry eye^*^	24	0
Infections and infestations		
Urinary tract infection	21	5
**Laboratory abnormality^†^**	**All grades, %**	**Grade 3–4, %**
Chemistry		
AST increased	75	5
Creatinine increased	71	3
Glucose increased	66	14
ALT increased	59	5
Sodium decreased	46	13
Phosphate decreased	44	9
Albumin decreased	39	2
Potassium decreased	26	5
Potassium increased	24	1
Calcium increased	21	1
Hematology		
Lymphocytes decreased	58	15
Hemoglobin decreased	53	7
Neutrophils decreased	30	9

Data reflect patients with urothelial cancer who received at least one dose of EV + Pembro from EV-302 (N = 440). Treatment consisted of EV 1.25 mg/kg (on days 1 and 8 of a 21-day cycle) and Pembro 200 mg (on day 1 of a 21-day cycle). Grading based on NCI CTCAE Version 4.03 (**Supplementary Table 2**) (35).

ALT, alanine aminotransferase; AST, aspartate aminotransferase; EV, enfortumab vedotin; EV + Pembro, enfortumab vedotin plus pembrolizumab combination; NCI CTCAE, National Cancer Institute Common Terminology Criteria for Adverse Events; Pembro, pembrolizumab.

^*^Includes multiple terms.

^†^The denominator used to calculate the rate for all grades and grade 3–4 varied from 407 to 439 based on the number of patients with a baseline value and at least one post-treatment value.

## 3 General management of AEs with EV + Pembro

In the authors’ experience, most AEs associated with the use of EV + Pembro may be managed or mitigated via early recognition of signs or symptoms and prompt medical intervention and/or use of dose modification(s). Education on AEs associated with the use of EV + Pembro, close monitoring, and collaboration with the patient and their caregivers, as well as involvement of a multidisciplinary healthcare team, are important to aid in prompt intervention and management.

Clinicians should also be mindful of existing AE management guidelines and clinical recommendations for both EV and Pembro as monotherapies, which include the use of dose modifications. As with many combination regimens, dose modifications may include dose holds (cycle delays, skipped doses), discontinuation of one or both drugs, or dose reductions of EV (dose reductions are not recommended for Pembro) to help manage treatment-emergent AEs. EV and Pembro both contribute to some of the AEs observed, such as skin reactions, pneumonitis, and diarrhea, which may make AE attribution and management more complex (22, 23). In the EV-302 and EV-103 trials, the study protocol provided recommended dose modifications to help manage treatment-emergent AEs (10, 25, 26). As AE attribution to one agent or the other may be difficult to assess with the combination, based on authors’ clinical experience, when AE attribution is not possible or is not yet known, an appropriate dose modification should be applied to both agents. When AE attribution is possible, recommended dose modifications should be applied to the related agent as clinically appropriate (dose modifications for EV and dose holds for Pembro). Additional details are included in the **Supplementary Appendix**, including EV dose modifications for EV-associated AEs (**Supplementary Table 3**), EV dose reduction schedule and dose re-escalation recommendations (**Supplementary Table 4**), and Pembro dose modifications for Pembro-associated AEs (**Supplementary Table 5**). Critically, in all cases where patients experience rapid onset, severe clinical presentation (grade ≥3), and/or worsening symptoms despite mitigation strategies, both drugs should be withheld until appropriate clinical assessments can be completed, the patient has received appropriate supportive care, and the AE has improved to grade ≤1.

ICIs such as Pembro are associated with immune-related AEs (irAEs) caused by off-target activation of the immune system that can involve any organ or tissue, further highlighting the importance of close monitoring for potential irAEs in patients receiving EV + Pembro (23, 30–32). Involvement of the skin, gastrointestinal tract, lungs, endocrine system, and musculoskeletal systems are relatively common with irAEs, while cardiovascular, hematologic, renal, neurologic, and ophthalmologic irAEs occur less frequently though may be more severe (30, 36). In many cases, irAEs can be managed with dose interruptions and/or supportive therapy, which may include the use of corticosteroids and/or immunosuppressants (37). With Pembro, dose reduction is not recommended, and treatment should be withheld until the AE has improved (**Supplementary Table 5**; **Supplementary Figure 1**) (23). Although irAEs can occur early in treatment, onset may be delayed and duration is prolonged (31, 32, 37, 38). Conversely, many AEs associated with EV typically arise within the first few weeks after initiation, although some cases can present months later (22).

## 4 AEs of clinical interest for EV and/or Pembro

### 4.1 Skin reactions

**Figure 1:**
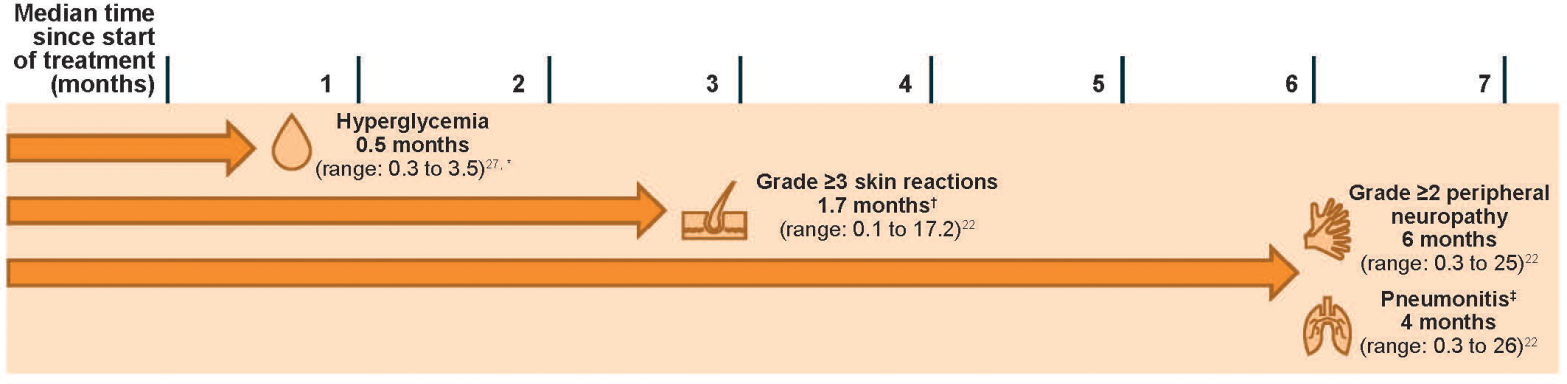
Median times to onset of select AEs in patients with la/mUC treated with EV + Pembro (N = 564). An AE may occur at any timepoint. Data reflect patients with urothelial cancer who received at least one dose of EV + Pembro from EV-302 and EV-103 (N = 564). Treatment consisted of EV 1.25 mg/kg (on days 1 and 8 of a 21-day cycle) and Pembro 200 mg (on day 1 of a 21-day cycle). Grading based on NCI CTCAE Version 4.03 (**Supplementary Table 2**) (35). AE, adverse event; EV, enfortumab vedotin; EV + Pembro, enfortumab vedotin plus pembrolizumab combination; la/mUC, locally advanced or metastatic urothelial cancer; NCI CTCAE, National Cancer Institute Common Terminology Criteria for Adverse Events; Pembro, pembrolizumab. ^*^Time to first onset for Dose Escalation Cohort/Cohort A (N = 45). Median onset for Cohort K was 0.53 months (N = 76) (26). ^†^Skin reactions of any grade may occur as early as the first cycle. ^‡^The EV US prescribing information uses the term “pneumonitis/interstitial lung disease” (22).

Skin reactions occur with EV and Pembro monotherapies (22, 23, 34), and were shown to occur more frequently with the combination therapy (22). In the pooled safety population of 564 patients who received EV + Pembro in EV-302 and EV-103, skin reactions (all grades) occurred in 70% of patients (22). The majority of the skin reactions that occurred with combination therapy were maculopapular rash, macular rash, and papular rash. Grade 3–4 skin reactions occurred in 17% of patients (grade 3: 16%; grade 4: 1%). A fatal reaction of bullous dermatitis occurred in one patient (0.2%) (22). The median time of onset for grade 3–4 skin reactions was 1.7 months (range, 0.1 to 17.2 months); notably, events occurred as early as the first cycle (**Figure 1**) (22). Of the patients who experienced a skin reaction and had data regarding resolution (N = 391), 59% had complete resolution. Of the patients with an ongoing skin reaction, 27% (43/159) were grade ≥2 at last follow-up (22). At a median follow-up of 4 years in the EV-103 Dose Escalation Cohort/Cohort A (n = 45), 90% of patients experiencing a skin reaction had improvement or resolution of symptoms at the last follow-up (**Supplementary Table 6**) (27).

Severe cutaneous adverse reactions such as Stevens–Johnson syndrome (SJS)/toxic epidermal necrolysis (TEN) have occurred with both EV and Pembro as monotherapies (22, 23). Therefore, it is important to educate patients about this potential risk and closely monitor patients for the emergence of any skin reaction, as SJS/TEN can be fatal. Patients should be instructed to immediately report the signs and symptoms of potential SJS/TEN, which include desquamating rash associated with malaise, fever ≥100.4°F (≥38°C), mucosal involvement (ocular, oral, genital), or dermatodynia (skin pain) (33).

#### 4.1.1 Management

**Figure 2:**
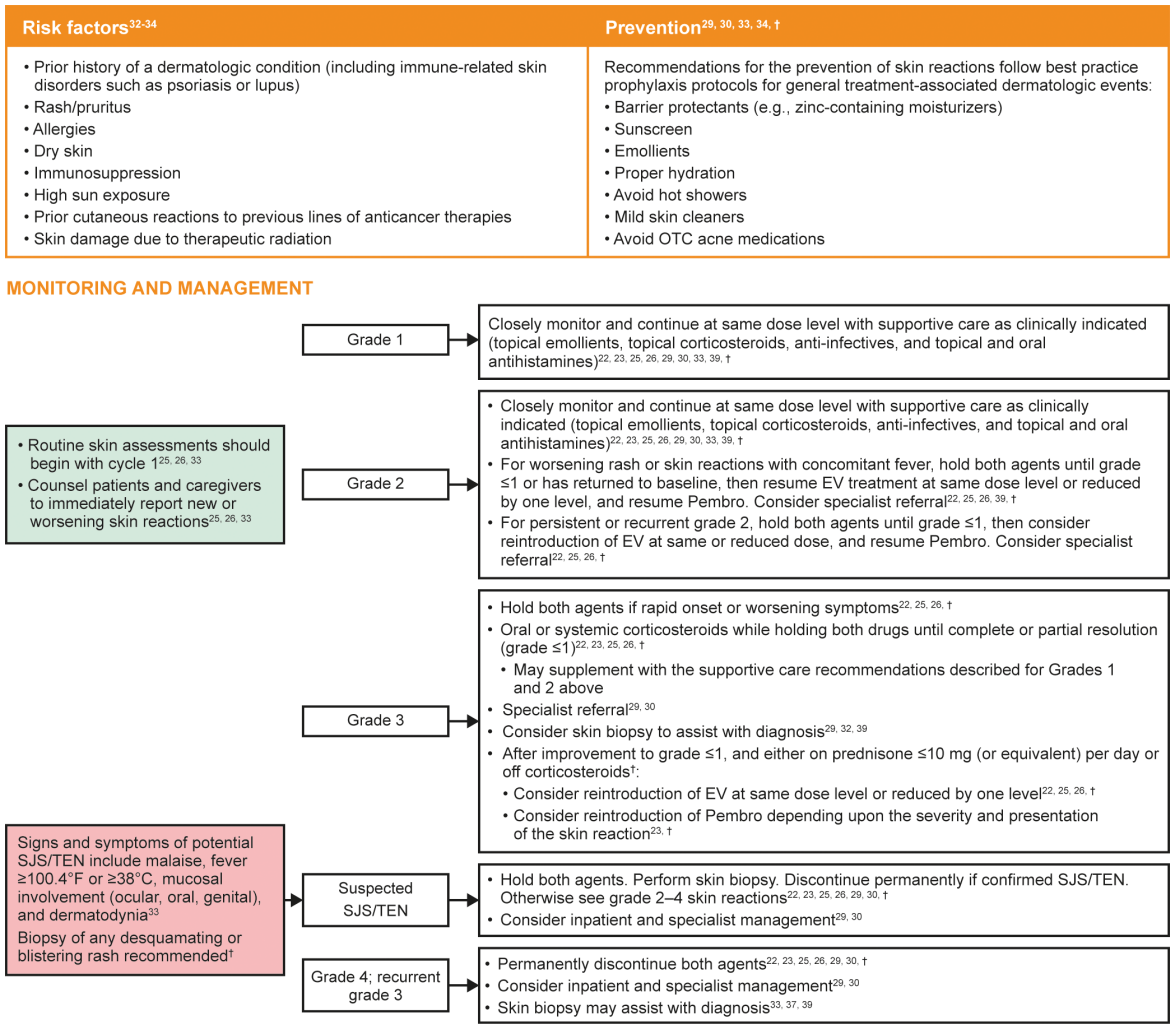
Risk factors and recommendations for the prevention, monitoring, and management of treatment-emergent skin reactions. Grading based on NCI CTCAE Version 4.03 (**Supplementary Table 2**) (35). EV, enfortumab vedotin; NCI CTCAE, National Cancer Institute Common Terminology Criteria for Adverse Events; OTC, over the counter; Pembro, pembrolizumab; SJS, Stevens–Johnson syndrome; TEN, toxic epidermal necrolysis. ^†^Recommendations based on clinical experience.

Risk factors and recommendations for the prevention, monitoring, and management of treatment-emergent skin reactions are shown in **Figure 2**. Although the presentation of EV-associated and Pembro-associated skin reactions can be similar, those associated with EV most commonly present in intertriginous, flexural, acral, and truncal areas, with skin that is often fragile, thin, and friable. Regular application of topical emollients, moisturizers, or barrier-protecting agents in these areas may be effective prophylaxis (33). EV-associated skin reactions can occur as early as the first cycle of treatment with EV (33) but may occur later. Conversely, Pembro-associated rashes often do not affect skin integrity, with maculopapular rashes and pruritus being the most frequent types associated with ICIs (30, 31). Skin reactions associated with ICIs may also occur within the first cycle (37), but in the authors’ collective experience these frequently occur later than those associated with EV. Clinical experience also shows that skin reactions that persist or worsen despite withholding both drugs are most likely immune-mediated, while EV-associated skin reactions may respond to dose holds more quickly as they are hypothesized to be due to direct cytotoxicity as a result of Nectin-4 expression in the skin (33, 39). Where attribution is unclear, dose holds of both agents should be considered. A thorough clinical exam, including inspection of the mucosa, and a biopsy can assist with diagnosing the type of skin reaction, including when reactions are refractory to both topical steroids and dose modifications, as well as identifying severe cutaneous adverse reactions such as SJS/TEN (33, 37, 39). Specialist referral is strongly recommended for skin reactions where diagnosis is unclear, those assessed as grade 3 or worse, those with associated blistering, or in instances where skin reactions are not responsive to topical steroids and/or dose modifications (22, 25, 26, 30).

Topical emollients, topical corticosteroids, and antihistamines are often adequate to treat mild to moderate non-exfoliative rashes (22, 23). Patients should be monitored closely for signs of improvement or worsening. Due to its more rapid elimination compared with Pembro (22, 23), reintroduction of EV may be considered at the same dose or reduced dose level with close monitoring for recurrence depending on the severity and presentation of the skin reaction. Restarting Pembro at its recommended dose at the 3-week dosing interval (23) can be considered depending upon the severity and presentation of the skin reaction. In cases of rapid onset or worsening symptoms, both therapies must be withheld and oral or systemic corticosteroids should be given until the skin reaction improves to grade ≤1 or resolution occurs. Reintroduction of either drug may be considered once patients only require low-dose steroids (≤10 mg prednisone or equivalent daily) or are off steroids to avoid masking a “flare up” skin event. If SJS/TEN is suspected, both therapies must be withheld immediately, and specialist consultation should be considered to confirm the diagnosis. If SJS/TEN is confirmed, both EV and Pembro should be permanently discontinued (22, 23, 33).

### 4.2 Peripheral neuropathy

Peripheral neuropathy is an anticipated AE associated with MMAE-containing ADCs (19, 40), and immune-mediated neuropathies have been known to occur rarely with Pembro. In the pooled safety set of patients treated with EV + Pembro, peripheral neuropathy was the second most common AE, occurring in 67% of patients (grade 3: 7%) and was the most frequent reason for EV discontinuation (22, 23). Onset of grade ≥2 peripheral neuropathy generally occurred later in the treatment course, with a median time of onset of 6 months (range, 0.3 to 25 months; **Figure 1**). Of the patients who experienced neuropathy and had data regarding resolution (N = 373), 13% had complete resolution, and 87% of patients had residual neuropathy at last follow-up. Of the patients with residual neuropathy at last evaluation, 45% (146/326) had grade ≥2 neuropathy (22). At a median follow-up of 4 years, nearly 70% of patients who had treatment-related peripheral neuropathy in EV-103 Dose Escalation/Cohort A had improvement or resolution of their symptoms at their last follow-up (**Supplementary Table 6**) (25, 27). The median time to resolution of any-grade peripheral neuropathy was 5.2 months (interquartile range, 3.5 to 8.6 months) (25).

#### 4.2.1 Management

**Figure 3:**
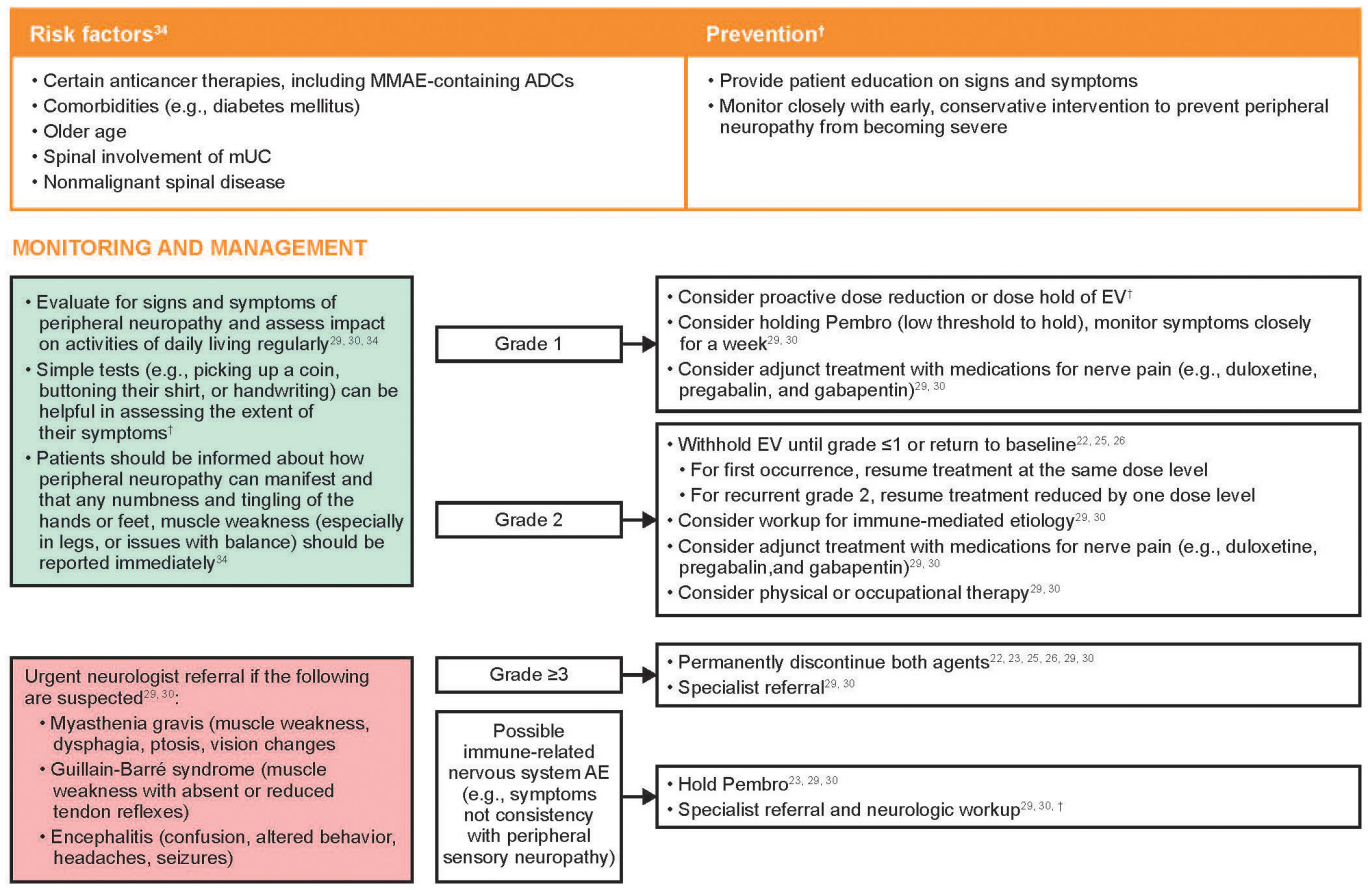
Risk factors and recommendations for the prevention, monitoring, and management of treatment-emergent peripheral neuropathy. Grading based on NCI CTCAE Version 4.03 (**Supplementary Table 2**) (35). ADC, antibody-drug conjugate; AE, adverse event; EV, enfortumab vedotin; MMAE, monomethyl auristatin E; mUC, metastatic urothelial cancer; NCI CTCAE, National Cancer Institute Common Terminology Criteria for Adverse Events; Pembro, pembrolizumab. ^†^Recommendations based on clinical experience.

Risk factors and recommendations for the prevention, monitoring, and management of treatment-emergent peripheral neuropathy are shown in **Figure 3**. Early recognition of treatment-emergent peripheral neuropathy and prompt intervention with appropriate use of dose modifications provides the best chance for resolution and may allow the patient to remain on therapy longer. As peripheral neuropathy is a cumulative AE, clinicians should be particularly aware of the potential for symptoms to develop as the duration of treatment increases. Patients may be reluctant to report signs and symptoms of peripheral neuropathy for fear of having their treatment interrupted or discontinued; however, patients should be educated that the use of dose modifications may help avoid worsening symptoms that could impact their activities of daily living or require treatment discontinuation. Patients should be informed that peripheral neuropathy can manifest as sensory and/or motor dysfunction, and any numbness and tingling of the hands or feet or muscle weakness should be reported quickly.

Peripheral sensory neuropathy is most often attributed to EV, and generally responsive to recommended EV dose holds or dose reductions. If neurological symptoms not consistent with peripheral sensory neuropathy are observed, referral to a neurologist is recommended for further neurologic workup, which may include magnetic resonance imaging of the spine and/or brain, lumbar puncture for cerebrospinal fluid analysis, electromyography, and nerve conduction studies (41). Neurological symptoms that should prompt an urgent referral to a neurologist include muscle weakness and/or paralysis, vision changes, ptosis, dysphagia, photophobia, confusion, and speech abnormalities, as these may be signs of serious neurological conditions such as myasthenia gravis, Guillain-Barré syndrome, or encephalitis (30, 31).

Adjunct treatment with medications typically used to treat nerve pain, such as duloxetine, pregabalin, and gabapentin, may provide benefit to some patients with painful peripheral sensory neuropathy (30, 31, 41). These drugs may take several weeks to take effect and patients should be counseled that they may not provide immediate relief.

Peripheral motor neuropathy, including muscle weakness, may also occur. Thorough musculoskeletal assessments at each clinical visit should be performed, including functional evaluation of fine motor skills, gait, and balance. Interventions including physical and occupational therapy may be considered, and mechanical aids (e.g., braces) may be used to alleviate issues with loss of balance and coordination (34).

### 4.3 Hyperglycemia/diabetes mellitus

Hyperglycemia and diabetic ketoacidosis, including fatal events, occurred in patients treated with both EV and Pembro as monotherapies. Hyperglycemia or the development of insulin-dependent diabetes is a rare irAE of ICIs (1.8%) and is thought to be caused by the autoimmune destruction of islet cells, similar to the process seen in type 1 diabetes mellitus; remission or resolution of ICI-induced diabetes following this destruction is rare (42). In contrast, although the pathophysiology of EV-induced hyperglycemia is not well understood, it can resolve. In clinical trials with EV monotherapy, 17% of patients developed hyperglycemia of any grade, while discontinuation due to hyperglycemia was limited to <1% of patients (22). In EV-302, hyperglycemia of any grade occurred in 13.0% of patients (as compared with 14% with EV monotherapy and 0.2% with Pembro monotherapy); grade 3–4 hyperglycemia occurred in 8.9% of patients. Hyperglycemia presented at a median onset time of approximately 2 weeks with both EV as monotherapy and in combination with Pembro (**Figure 1**) (22, 23). In the EV-103 Dose Escalation Cohort/Cohort A (n = 45), all patients who experienced hyperglycemia had improvement or resolution of their hyperglycemia at their last follow-up, with a median time to resolution of 1.6 months (interquartile range, 0.7 to 1.6 months) (**Supplementary Table 6**) (25, 27). In this cohort, hyperglycemia occurred more frequently in patients with a body mass index of ≥30 kg/m^2^ or with baseline hyperglycemia or diabetes mellitus (25), a trend also observed in a study evaluating EV monotherapy (18, 22).

#### 4.3.1 Management

**Figure 4:**
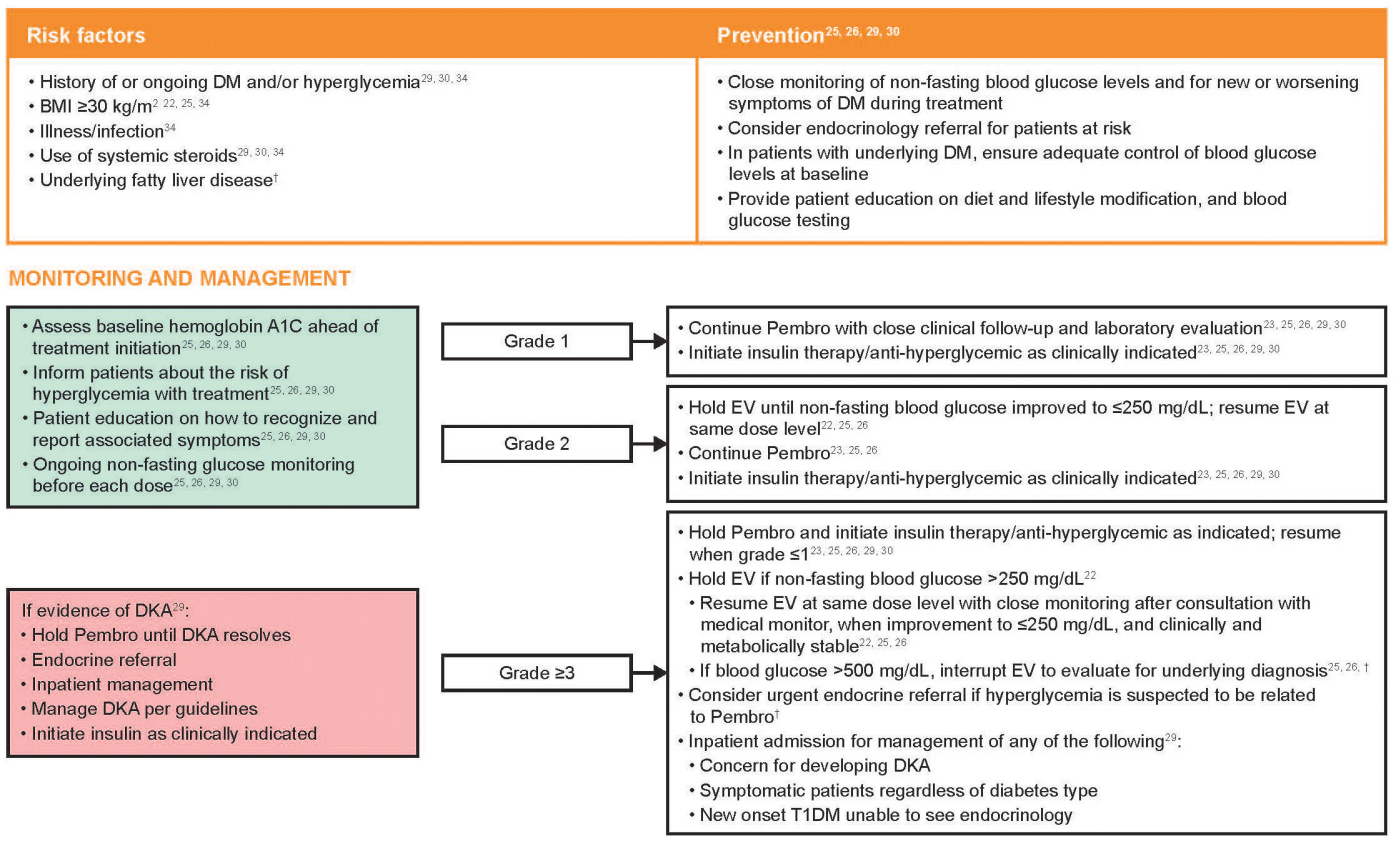
Risk factors and recommendations for the prevention, monitoring, and management of treatment-emergent hyperglycemia. Grading based on NCI CTCAE Version 4.03 (**Supplementary Table 2**) (35). BMI, body mass index; DKA, diabetic ketoacidosis; DM, diabetes mellitus; EV, enfortumab vedotin; NCI CTCAE, National Cancer Institute Common Terminology Criteria for Adverse Events; Pembro, pembrolizumab; T1DM, type 1 diabetes mellitus. ^†^Recommendations based on clinical experience.

Risk factors and recommendations for the prevention, monitoring, and management of treatment-emergent hyperglycemia are shown in **Figure 4**. Other etiologies for hyperglycemia, such as infection or systemic corticosteroids, should also be considered (23, 30). If there is evidence of ketosis or insulin insufficiency and/or resistance, urgent endocrine consultation is recommended; both agents should be withheld in the setting of ketosis or grade ≥3 hyperglycemia (29, 30). The development of autoimmune diabetes may be accompanied by reduced C-peptide levels as well as the presence of GAD65 and islet cell antibodies (31, 42), which would not be present with steroid use, infection, or EV-associated hyperglycemia. EV should be withheld if non-fasting blood glucose is >250 mg/dL; once the blood glucose has improved to ≤250 mg/dL and the patient is clinically and metabolically stable, EV can be resumed at the same level. Non-fasting blood glucose should be re-tested before each EV dose (22, 26).

### 4.4 Pneumonitis

Pneumonitis, including severe, life-threatening or fatal events, occurred in patients treated with both EV and Pembro as monotherapies (3% and 3.4%, respectively), and occurred at higher rates when given as combination therapy (22, 23). In the pooled safety population, pneumonitis occurred in 10% of patients treated with EV + Pembro (grade ≥3 in 4%; fatal in two patients [0.4%]). Median time to onset of any grade pneumonitis was 4 months (range, 0.3 to 26 months; **Figure 1**) (22).

#### 4.4.1 Management

**Figure 5:**
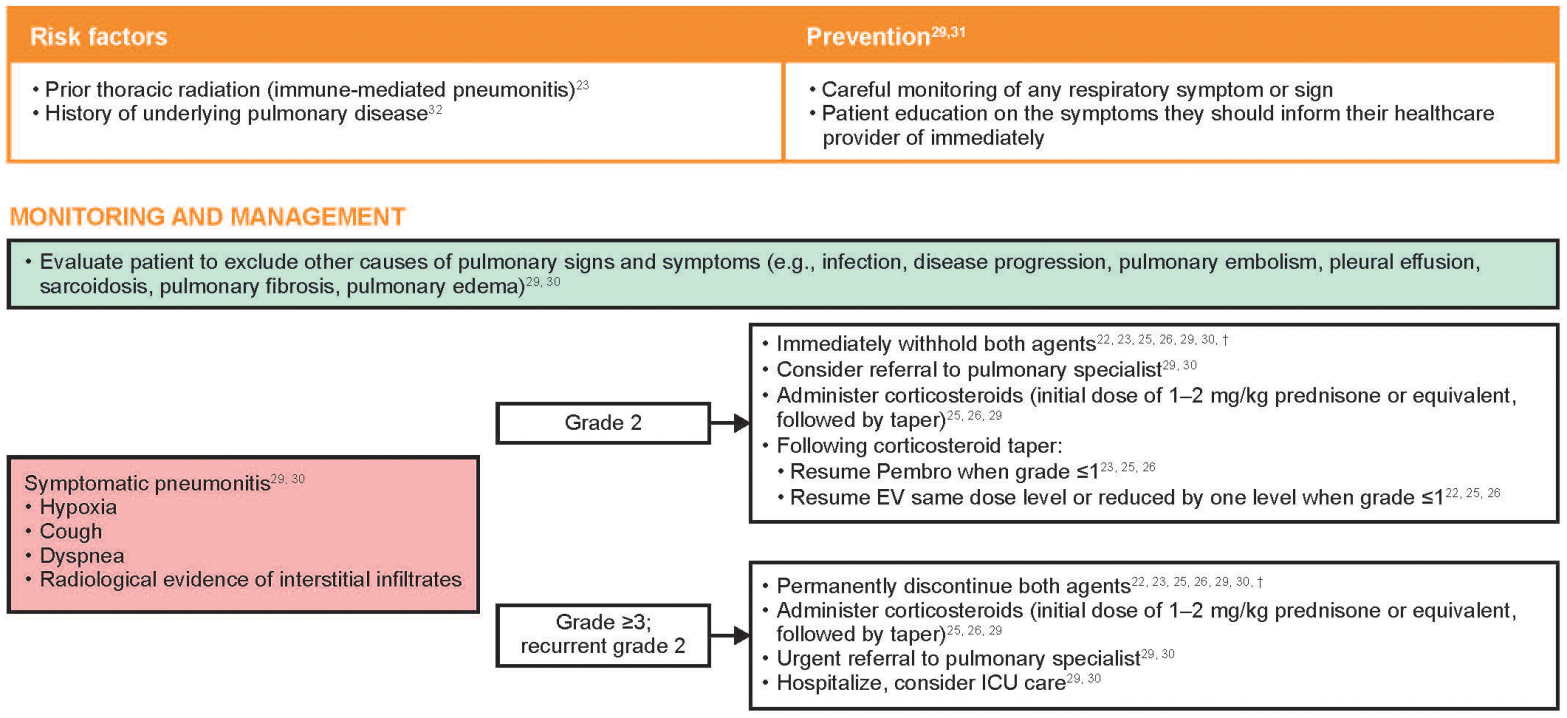
Risk factors and recommendations for the prevention, monitoring, and management of treatment-emergent pneumonitis^*^. Grading based on NCI CTCAE Version 4.03 (**Supplementary Table 2**) (35). EV, enfortumab vedotin; ICU, intensive care unit; NCI CTCAE, National Cancer Institute Common Terminology Criteria for Adverse Events; Pembro, pembrolizumab. ^*^The EV US prescribing information uses the term “pneumonitis/interstitial lung disease” (22). ^†^Recommendations based on clinical experience.

Risk factors and recommendations for the prevention, monitoring, and management of treatment-emergent pneumonitis are shown in **Figure 5**. In the event of symptomatic pneumonitis, both therapies should be immediately withheld. Use of corticosteroids, immunosuppressive agents, and supportive care should be provided as clinically indicated, and a referral to a pulmonary specialist should be considered. In the author’s experience, biopsy or bronchoscopy may be considered to rule out infection or other etiologies of respiratory symptoms, but the diagnostic evaluation should not significantly delay the prompt initiation of corticosteroids in a symptomatic patient. In patients with grade 2 pneumonitis, Pembro should be withheld until symptoms are partially or completely resolved (grade 1 or 0) after corticosteroid taper. EV should also be withheld until grade ≤1, at which point treatment can be resumed at the same dose level or reduced by one dose level. For patients in whom the causal agent is undetermined, once symptoms of pneumonitis resolve, EV should generally be reinitiated first along with close monitoring, followed later by Pembro. In patients with grade 3–4 pneumonitis, EV and Pembro should both be permanently discontinued. Radiographic evidence of improvement may lag weeks or months after clinical improvement is observed; therefore, radiographic improvement need not be a prerequisite for restarting treatment.

### 4.5 Gastrointestinal events

Gastrointestinal events occurred frequently in patients treated with EV + Pembro in the EV-302 study, including diarrhea (38%), constipation (26%), nausea (26%), and dysgeusia (21%). While most events were mild in nature, 3.6% had severe diarrhea (grade ≥3) (22, 23). In EV-103 Cohort K, patients who received EV + Pembro reported treatment-emergent diarrhea that initially worsened at week 3 but resolved at week 8 and week 24 (43).

#### 4.5.1 Management

Risk factors and recommendations for the prevention, monitoring, and management of treatment-emergent gastrointestinal events are shown in **Table 2**. As diarrhea is a known AE that occurs with both EV and immunotherapy, it is important to monitor for symptoms and assess fluid and electrolyte status (22, 29). Diarrhea in conjunction with abdominal pain, rectal bleeding, mucus in the stool, and fever should prompt a referral to a gastrointestinal specialist to rule out colitis, a potentially serious irAE (29, 30); steroids can be started empirically during work-up if clinically indicated. For uncomplicated diarrhea, oral hydration, dietary modifications, and over-the-counter anti-diarrheals/anti-emetics should be utilized for symptom management. Based on clinical experience, diarrhea that transiently worsens over the first few cycles then subsequently improves is likely attributed to EV. In contrast, diarrhea that persists and/or worsens over time is most likely immune-mediated and associated with Pembro. Diarrhea that does not respond to high-dose corticosteroids may require the use of immunosuppressive treatments. Stool studies may also be performed to rule out infection and test for inflammatory markers, including stool lactoferrin and calprotectin (29).

**Table 2:** Risk factors and recommendations for the prevention, monitoring, and management of other treatment-emergent AEs.

	Risk factors	Prevention	Monitoring	Management
Gastrointestinal events	• Immunotherapy (34)	• Ensure adequate nutrition and hydration • Avoiding foods with strong smells • Small, frequent meals (34)	• Assess fluid and electrolyte status (34) • Patient education on the symptoms they should inform their healthcare provider of immediately (34) • Diarrhea in conjunction with abdominal pain, rectal bleeding, mucus in the stool, and fever should prompt work-up for colitis (29, 30)	**Vomiting/diarrhea/colitis** • Grade 1 • Oral hydration, dietary modifications (29) • OTC anti-diarrheals^†^ • Anti-emetics as needed^†^ • Grade 2 (25, 26) • Hold Pembro; continue EV at same dose • Consider corticosteroids if suspected to be immune-related (initial dose of 1–2 mg/kg prednisone or equivalent) followed by taper • Reintroduce Pembro following taper if diarrhea improves to grade ≤1 • Grade 3 • Hold Pembro; for recurrent grade 3, permanently discontinue Pembro (25, 26) • Corticosteroids as above (25, 26) • Hold EV until grade ≤1, then resume at same dose or consider dose reduction by one level • For persistent immune-mediated diarrhea, consider IV corticosteroids or infliximab; hospitalize if indicated (29, 30) • Grade 4 • Permanently discontinue both drugs (25, 26) • Corticosteroids as above (25, 26) • For persistent immune-mediated diarrhea, consider IV corticosteroids or infliximab; hospitalize if indicated (29, 30) • If vomiting/diarrhea improves to grade ≤2 within 72 hours with supportive management, no discontinuation of EV needed (25, 26)
Fatigue	• Comorbidities (disease-related or treatment-related fatigue) • Anemia • Anorexia • Weight loss • Endocrinopathies (44)	–	• Evaluate patient for any other known irAEs that can commonly manifest as fatigue (e.g., adrenal insufficiency, hypophysitis, and hypothyroidism) (29, 30)	• Non-pharmacological interventions (44) • Lifestyle changes (44) • Consider short-term pharmaceutical interventions (44) • Dose interruption/modification, particularly EV^†^
Ocular disorders	• Older age (for dry eyes) • Use of anticancer therapies such as ADCs (for keratitis and corneal ulcerations) • Contact lens use (for developing keratitis) (34)	• Practice good hygiene around the eyes • Artificial tear drops (34)	• Patient education on the symptoms that they should inform their healthcare provider of immediately	• Any ocular symptoms and/or changes in vision should prompt a referral to an ophthalmologist (25, 26) • Consider dose interruption or reduction of EV for symptomatic ocular disorders (22)^†^ • For grade 2 corneal AEs, hold EV until grade ≤1 or return to baseline, then resume at same dose level (25, 26) • Dose holds for Pembro (29): • Grade ≥3 uveitis • Grade ≥2 scleritis • Grade ≥2 episcleritis • Cool compression (dry eye)^†^ • Warm compression (blepharitis) (30) • Lubricating ointment, artificial tears (34) • Corticosteroid eye drops (34) • Avoid use of contacts (29, 34)

Grading based on NCI CTCAE Version 4.03 (**Supplementary Table 2**) (35).

ADC, antibody-drug conjugate; AE, adverse event; EV, enfortumab vedotin; irAE, immune-mediated adverse event; IV, intravenous; NCI CTCAE, National Cancer Institute Common Terminology Criteria for Adverse Events; OTC, over the counter; Pembro, pembrolizumab.

^†^Recommendations based on clinical experience.

The recommended management of nausea and vomiting with the treatment combination is the same as for either EV or Pembro monotherapies, with prompt intervention and/or prophylaxis used to reduce the risk of complications associated with dehydration such as acute kidney failure and/or deterioration of poor pre-existing renal function.

### 4.6 Fatigue

Generalized fatigue is common in patients with cancer, which can be multifactorial (e.g., related to disease, treatment, anemia, anorexia, weight loss, endocrinopathy, or other factors) (44–47). In patients treated with EV + Pembro in EV-302, 51% of patients experienced fatigue, with 6% experiencing grade 3–4 fatigue (22, 23). In EV-103 Cohort K, patients who received EV + Pembro reported treatment-emergent fatigue that initially worsened at week 3 but resolved at week 8 and week 18 (43).

#### 4.6.1 Management

Risk factors and recommendations for the prevention, monitoring, and management of treatment-emergent fatigue are shown in **Table 2**. Patient education and counseling are central to the effective management of fatigue (44–47). Patients should be reassured that treatment-related fatigue is not necessarily an indicator of disease progression (44). Fatigue can have a substantial impact on patients’ quality of life, therefore the approach to management should consider the patient holistically. Depending on the etiology of the fatigue, nonpharmacologic interventions may be beneficial. If diagnostic evaluation of fatigue indicates hypothyroidism and/or adrenal insufficiency as the cause (e.g., morning adrenocorticotropic hormone and corticotrophin-releasing hormone tests), suggesting an immune-mediated etiology, appropriate referral (e.g., endocrinology) and management should be initiated. In patients with grade ≥3 fatigue, EV should be withheld. EV may be reintroduced at the same dose or a reduced dose once symptoms improve to grade ≤1 (26). Consider withholding Pembro if the fatigue has not improved by the next cycle.

### 4.7 Ocular disorders

In patients treated with EV + Pembro in EV-302, the most common ocular disorder was dry eye (24%, as compared with 40% with EV monotherapy and <1% with ICIs) and was generally mild (22, 23, 31).

#### 4.7.1 Management

Risk factors and recommendations for the prevention, monitoring, and management of treatment-emergent ocular reactions are shown in **Table 2**. Recommendations for symptomatic management may include cool compresses over closed eyes for dry eye, warm compresses for conjunctivitis or blepharitis, lubricating ointment or artificial tears, and corticosteroid eye drops as clinically indicated. Referral to an ophthalmologist is recommended for diagnosis and management of ocular disorders, when feasible. Consider dose interruption or dose reduction of EV for symptomatic ocular disorders (22). Dose holds for Pembro may be indicated in some cases (such as with grade ≥3 uveitis, grade ≥2 scleritis, and grade ≥2 episcleritis (29).

## 5 Patient education

In the authors’ experience, patients may be more forthcoming with information on their treatment experience when they understand that dose modifications are a normal part of receiving cancer treatment and were used in the clinical trials to manage AEs. Patients may minimize or ignore what they perceive to be mild reactions or withhold information out of concern that therapy may be interrupted or discontinued by their treating physician/team. Therefore, HCPs should emphasize to their patients that prompt management of AEs via dose modifications may allow them to stay on therapy longer. It is important to encourage patients and their caregivers to report anything out of the ordinary so that the care team can determine the seriousness of the AE and whether additional care may be needed. In addition, some patients may not proactively report issues being addressed by their primary care physician or other providers, highlighting the importance of encouraging the patient to follow up with their oncology care team after other visits.

Prior to initiating treatment with EV + Pembro, it is important to manage patient expectations through clear discussions of treatment benefits and risks as related to known potential AEs. Patient education should place emphasis on AEs of clinical interest, especially those that may occur at any time, may take time to resolve (e.g., peripheral sensory neuropathy), or may not resolve. The patient information documents for EV and for Pembro from the respective US prescribing information labels should be provided to patients as a resource (22, 23). Patients should be counselled to report any new and/or worsening symptoms to their care team to determine whether prompt intervention is necessary (**Supplementary Table 7**). Frequent, regular engagement and communication with patients and their caregivers are critical to enable timely identification and appropriate triage of AEs and coordinate care with other healthcare providers. Every visit where EV + Pembro is administered creates an opportunity to ask about and assess AEs (**Supplementary Table 7**). Mobile applications that can track side effects in real time, such as the ASCO Cancer.Net mobile application, can also be valuable tools for patient follow-up and detection of potentially more serious AEs (48).

Where possible, information should be conveyed both verbally and provided in written form. Patient education fact cards can be used to remind patients of potential AEs, self-care measures, medication information, scheduling, important contact information, and when to contact the care team. When warranted, oncologists should consider connecting patients with other specialists who may be able to help manage AEs, such as dermatologists, neurologists, gastroenterologists, endocrinologists, pulmonologists, or ophthalmologists. Patient advocacy groups, such as the Bladder Cancer Advocacy Network, also have extensive resources available for patients with bladder cancer.

## 6 Conclusions

Education on AEs associated with the use of EV + Pembro, proactive monitoring, assessment and management of AEs may help minimize toxicity and maximize clinical benefit and patient experience with EV + Pembro. A partnership between HCPs and patients and their caregivers, frequent communication, and upfront patient education are key in identifying emerging AEs. Factors such as the onset and characteristics of AEs and certain diagnostic steps may help the oncology care team determine attribution of AEs and appropriate clinical management, including dose modifications, supportive measures, and other interventions. As demonstrated in EV-302, EV + Pembro nearly doubled both median OS and median PFS in patients with previously untreated la/mUC regardless of cisplatin-eligibility and had a safety profile consistent with that observed in EV-103. Additional data from EV-302, as well as ongoing clinical trials in muscle invasive bladder cancer (EV-303/KN-905 [NCT03924895]; EV-304/KN-B15 [NCT04700124]), are expected to provide additional insight into best practices for minimizing toxicity and maximizing patient experience and clinical benefit with this novel combination.

## Author contributions

BB: Writing – original draft, Writing – review & editing. AM: Writing – original draft, Writing – review & editing. HA: Writing – original draft, Writing – review & editing. CE: Writing – original draft, Writing – review & editing. IB: Writing – original draft, Writing – review & editing. JR: Writing – original draft, Writing – review & editing. MM: Writing – original draft, Writing – review & editing.
