# Supplementary material for: Managing potential adverse events during treatment with enfortumab vedotin + pembrolizumab in patients with advanced urothelial cancer
Source: Front Oncol. 2024 Apr 22;14:1326715. doi: 10.3389/fonc.2024.1326715 (PMC11071165; doi:10.3389/fonc.2024.1326715)
Supplement: Supplementary file 1 [file DataSheet_1.pdf]

Supplementary Table 1. AEs of clinical interest leading to dose modifications ≥2% in patients treated with EV + Pembro in EV-302 (N = 440) [1, 2].

	Dose modifications for EV only			Dose modifications for Pembro only†	
	**Dose interruption*, %**	**Dose reduction, %**	**Dose discontinuation, %**	**Dose interruption*, %**	**Dose discontinuation, %**
Skin reactions	16	16	4.1	17	3.4
Peripheral neuropathy	22	13	15	7	–
Hyperglycemia	3.6	–	–	2.5	–
Diarrhea	5	–	–	4.3	–
Fatigue	3.9	2.7	–	3	–
Pneumonitis/ILD	4.8	–	2.3	3.6	4.8
Ocular disorders	0	0	0	0	0

AE, adverse event; EV, enfortumab vedotin; EV + Pembro, enfortumab vedotin plus pembrolizumab combination; ILD, interstitial lung disease; Pembro, pembrolizumab.

*Dose interruption indicates a skipped dose or a cycle delay.

†No dose reduction for Pembro is recommended per the prescribing information [2]. Data reflect patients with urothelial cancer who received at least one dose of EV + Pembro from EV-302 (N = 440). Treatment consisted of EV 1.25 mg/kg (on days 1 and 8 of a 21-day cycle) and Pembro 200 mg (on day 1 of a 21-day cycle).

Supplementary Table 2. Severity grading for AEs of clinical interest based on NCI CTCAE Version 4.03 [3].

Grade refers to the severity of the AE. The CTCAE displays Grades 1 through 5 with unique clinical descriptions of severity for each AE based on this general guideline:

- Grade 1: Mild; asymptomatic or mild symptoms; clinical or diagnostic observations only; intervention not indicated
- Grade 2: Moderate; minimal, local, or noninvasive intervention indicated; limiting age-appropriate instrumental ADL[*]
    - Instrumental ADL refer to preparing meals, shopping for groceries or clothes, using the telephone, managing money, etc
- Grade 3: Severe or medically significant but not immediately life-threatening; hospitalization or prolongation of hospitalization indicated; disabling; limiting self-care ADL[b]
    - Self-care ADL refer to bathing, dressing and undressing, feeding self, using the toilet, taking medications, and not bedridden
- Grade 4: Life-threatening consequences; urgent intervention indicated
- Grade 5: Death related to AE
- A Semi-colon indicates “or” within the description of the grade
- A single dash (–) indicates a grade is not available

	Grade					
**AE**	**Definition**	**1**	**2**	**3**	**4**	**5**
**Eye disorders**						
Blurred vision	A disorder characterized by visual perception of unclear or fuzzy images	Intervention not indicated	Symptomatic; limiting instrumental ADL	Limiting self-care ADL	–	–
Conjunctivitis	A disorder characterized by inflammation, swelling, and redness to the conjunctiva of the eye	Asymptomatic or mild symptoms; intervention not indicated	Symptomatic; topical intervention indicated (e.g., antibiotics); limiting instrumental ADL	Limiting self-care ADL	–	–
Dry eye	A disorder characterized by dryness of the cornea and conjunctiva	Asymptomatic; clinical or diagnostic observations only; mild symptoms relieved by lubricants	Symptomatic; multiple agents indicated; limiting instrumental ADL	Decrease in visual acuity (<20/40); limiting self-care ADL	–	–
Uveitis	A disorder characterized by inflammation to the uvea of the eye	Asymptomatic; clinical or diagnostic observations only	Anterior uveitis; medical intervention indicated	Posterior or pan-uveitis	Blindness (20/200 or worse) in the affected eye	–
Scleritis	A disorder characterized by involvement of the sclera of the eye	Asymptomatic; clinical or diagnostic observations only	Symptomatic, limiting instrumental ADL; moderate decrease in visual acuity (20/40 or better)	Symptomatic, limiting self-care ADL; marked decrease in visual acuity (worse than 20/40)	Blindness (20/200 or worse) in the affected eye	–
Eye disorders – Other specify	N/A	Asymptomatic or mild symptoms; clinical or diagnostic observations only; intervention not indicated	Moderate; minimal, local or noninvasive intervention indicated; limiting age appropriate instrumental ADL	Severe or medically significant but not immediately sight threatening; hospitalization or prolongation of existing hospitalization indicated; disabling; limiting self-care ADL	Sight-threatening consequences; urgent intervention indicated; blindness (20/200 or worse) in the affected eye	–

Gastrointestinal disorders						
Colitis	A disorder characterized by inflammation of the colon	Asymptomatic; clinical or diagnostic observations only; intervention not indicated	Abdominal pain; mucus or blood in stool	Severe abdominal pain; change in bowel habits; medical intervention indicated; peritoneal signs	Life-threatening consequences; urgent intervention indicated	Death
Constipation	A disorder characterized by irregular and infrequent or difficult evacuation of the bowels	Occasional or intermittent symptoms; occasional use of stool softeners, laxatives, dietary modification, or enema	Persistent symptoms with regular use of laxatives or enemas; limiting instrumental ADL	Obstipation with manual evacuation indicated; limiting self-care ADL	Life-threatening consequences; urgent intervention indicated	Death
Diarrhea	A disorder characterized by frequent and watery bowel movements	Increase of <4 stools per day over baseline; mild increase in ostomy output compared to baseline	Increase of 4–6 stools per day over baseline; moderate increase in ostomy output compared to baseline	Increase of ≥7 stools per day over baseline; incontinence; hospitalization indicated; severe increase in ostomy output compared to baseline; limiting self-care ADL	Life-threatening consequences; urgent intervention indicated	Death
Nausea	A disorder characterized by a queasy sensation and/or the urge to vomit	Loss of appetite without alteration in eating habits	Oral intake decreased without significant weight loss, dehydration, or malnutrition	Inadequate oral caloric or fluid intake; tube feeding, TPN or hospitalization indicated	–	–
Vomiting	A disorder characterized by the reflexive act of ejecting the contents of the stomach through the mouth	1–2 episodes (separated by 5 minutes) in 24 hrs	3–5 episodes (separated by 5 minutes) in 24 hrs	≥6 episodes (separated by 5 minutes) in 24 hrs; tube feeding, TPN, or hospitalization indicated	Life-threatening consequences; urgent intervention indicated	Death

**General disorders and administration site conditions**						
Fatigue	A disorder characterized by a state of generalized weakness with a pronounced inability to summon sufficient energy to accomplish daily activities	Fatigue relieved by rest	Fatigue not relieved by rest; limiting instrumental ADL	Fatigue not relieved by rest, limiting self-care ADL	–	–
Peripheral edema (CTCAE term: “edema limbs”)	A disorder characterized by swelling due to excessive fluid accumulation in the upper or lower extremities	5–10% inter-limb discrepancy in volume or circumference at point of greatest visible difference; swelling or obscuration of anatomic architecture on close inspection	>10–30% inter-limb discrepancy in volume or circumference at point of greatest visible difference; readily apparent obscuration of anatomic architecture; obliteration of skin folds; readily apparent deviation from normal anatomic contour; limiting instrumental ADL	>30% inter-limb discrepancy in volume; gross deviation from normal anatomic contour; limiting self-care ADL	–	–
**Investigations**						
Decreased weight (CTCAE term: “weight loss”)	A finding characterized by a decrease in overall body weight; for pediatrics, less than the baseline growth curve	5 to <10% from baseline; intervention not indicated	10 to <20% from baseline; nutritional support indicated	≥20% from baseline; tube feeding or TPN indicated	–	–
**Metabolism and nutrition disorders**						
Decreased appetite (CTCAE term: “anorexia”)	A disorder characterized by a loss of appetite	Loss of appetite without alteration in eating habits	Oral intake altered without significant weight loss or malnutrition; oral nutritional supplements indicated	Associated with significant weight loss or malnutrition (e.g., inadequate oral caloric and/or fluid intake); tube feeding or TPN indicated	Life-threatening consequences; urgent intervention indicated	Death

Hyperglycemia	A disorder characterized by laboratory test results that indicate an elevation in the concentration of blood sugar. It is usually an indication of diabetes mellitus or glucose intolerance	Fasting glucose value >ULN–160 mg/dL; Fasting glucose value >ULN–8.9 mmol/L	Fasting glucose value >160–250 mg/dL; Fasting glucose value >8.9–13.9 mmol/L	>250–500 mg/dL; >13.9–27.8 mmol/L; hospitalization indicated	>500 mg/dL; >27.8 mmol/L; life-threatening consequences	Death
**Musculoskeletal and connective tissue disorders**						
Arthralgia	A disorder characterized by a sensation of marked discomfort in a joint	Mild pain	Moderate pain; limiting instrumental ADL	Severe pain; limiting self-care ADL	–	–
**Nervous system disorders**						
Dizziness	A disorder characterized by a disturbing sensation of lightheadedness, unsteadiness, giddiness, spinning, or rocking	Mild unsteadiness or sensation of movement	Moderate unsteadiness or sensation of movement; limiting instrumental ADL	Severe unsteadiness or sensation of movement; limiting self-care ADL	–	–
Dysesthesia	A disorder characterized by distortion of sensory perception, resulting in an abnormal and unpleasant sensation	Mild sensory alteration	Moderate sensory alteration; limiting instrumental ADL	Severe sensory alteration; limiting self-care ADL	–	–
Dysgeusia	A disorder characterized by abnormal sensual experience with the taste of foodstuffs; it can be related to a decrease in the sense of smell	Altered taste but no change in diet	Altered taste with change in diet (e.g., oral supplements); noxious or unpleasant taste; loss of taste	–	–	–
Gait disturbance	A disorder characterized by walking difficulties	Mild change in gait (e.g., wide-based, limping, or hobbling)	Moderate change in gait (e.g., wide-based, limping, or hobbling); assistive device	Disabling; limiting self-care ADL	–	–

			indicated; limiting instrumental ADL			
Muscular weakness	A disorder characterized by a reduction in the strength of muscles in multiple anatomic sites	Symptomatic; weakness perceived by patient but not evident on physical exam	Symptomatic; weakness evident on physical exam; weakness limiting instrumental ADL	Weakness limiting self-care ADL; disabling	–	–
Neuralgia	A disorder characterized by intense painful sensation along a nerve or group of nerves	Mild pain	Moderate pain; limiting instrumental ADL	Severe pain; limiting self-care ADL	–	–
Paresthesia	A disorder characterized by functional disturbances of sensory neurons resulting in abnormal cutaneous sensations of tingling, numbness, pressure, cold, and warmth that are experienced in the absence of a stimulus	Mild symptoms	Moderate symptoms; limiting instrumental ADL	Severe symptoms; limiting self-care ADL	–	–
Peripheral motor neuropathy	A disorder characterized by inflammation or degeneration of the peripheral motor nerves	Asymptomatic; clinical or diagnostic observations only; intervention not indicated	Moderate symptoms; limiting instrumental ADL	Severe symptoms; limiting self-care ADL; assistive device indicated	Life-threatening consequences; urgent intervention indicated	Death
Peripheral sensory neuropathy	A disorder characterized by inflammation or degeneration of the peripheral sensory nerves	Asymptomatic; loss of deep tendon reflexes or paresthesia	Moderate symptoms; limiting instrumental ADL	Severe symptoms; limiting self-care ADL	Life-threatening consequences; urgent intervention indicated	Death
**Respiratory, thoracic, and mediastinal disorders**						
Pneumonitis	A disorder characterized by inflammation focally or diffusely affecting the lung parenchyma	Asymptomatic; clinical or diagnostic observations only; intervention not indicated	Symptomatic; medical intervention indicated; limiting instrumental ADL	Severe symptoms; limiting self-care ADL; oxygen indicated	Life-threatening respiratory compromise; urgent intervention indicated	Death

					(e.g., tracheotomy or intubation)	
**Skin and subcutaneous tissue disorders**						
Alopecia	A disorder characterized by a decrease in density of hair compared to normal for a given individual at a given age and body location	Hair loss of <50% of normal for that individual that is not obvious from a distance but only on close inspection; a different hair style may be required to cover the hair loss but it does not require a wig or hair piece to camouflage	Hair loss of ≥50% normal for that individual that is readily apparent to others; a wig or hair piece is necessary if the patient desires to completely camouflage the hair loss; associated with psychosocial impact	–	–	–
Bullous dermatitis	A disorder characterized by inflammation of the skin characterized by the presence of bullae which are filled with fluid	Asymptomatic; blisters covering <10% BSA	Blisters covering 10–30% BSA; painful blisters; limiting instrumental ADL	Blisters covering >30% BSA; limiting self-care ADL	Blisters covering >30% BSA; associated with fluid or electrolyte abnormalities; ICU care or burn unit indicated	Death
Dry skin	A disorder characterized by flaky and dull skin; the pores are generally fine; the texture is a papery thin texture.	Covering <10% BSA and no associated erythema or pruritus	Covering 10–30% BSA and associated with erythema or pruritus; limiting instrumental ADL	Covering >30% BSA and associated with pruritus; limiting self-care ADL	–	–
Erythema multiforme	A disorder characterized by target lesions (a pink-red ring around a pale center)	Target lesions covering <10% BSA and not associated with skin tenderness	Target lesions covering 10–30% BSA and associated with skin tenderness	Target lesions covering >30% BSA and associated with oral or genital erosions	Target lesions covering >30% BSA; associated with fluid or electrolyte abnormalities; ICU care or burn unit indicated	Death
Palmar-plantar erythrodysesthesia syndrome	A disorder characterized by redness, marked discomfort, swelling, and tingling in the palms of	Minimal skin changes or dermatitis (e.g., erythema, edema, or	Skin changes (e.g., peeling, blisters, bleeding, edema, or hyperkeratosis) with	Severe skin changes (e.g., peeling, blisters, bleeding, edema, or hyperkeratosis) with	–	–

	the hands or the soles of the feet	hyperkeratosis) without pain	pain; limiting instrumental ADL	pain; limiting self-care ADL		
Pruritus	A disorder characterized by an intense itching sensation	Mild or localized; topical intervention indicated	Intense or widespread; intermittent; skin changes from scratching (e.g., edema, papulation, excoriations, lichenification, oozing/crusts); oral intervention indicated; limiting instrumental ADL	Intense or widespread; constant; limiting self-care ADL or sleep; oral corticosteroid or immunosuppressive therapy indicated	–	–
Rash maculo-papular	A disorder characterized by the presence of macules (flat) and papules (elevated). Also known as morbilliform rash, it is one of the most common cutaneous adverse events, frequently affecting the upper trunk, spreading centripetally and associated with pruritus	Macules/papules covering <10% BSA with or without symptoms (e.g., pruritus, burning, tightness)	Macules/papules covering 10–30% BSA with or without symptoms (e.g., pruritus, burning, tightness); limiting instrumental ADL	Macules/papules covering >30% BSA with or without associated symptoms; limiting self-care ADL	–	–
Stevens–Johnson syndrome	A disorder characterized by less than 10% total body skin area separation of dermis. The syndrome is thought to be a hypersensitivity complex affecting the skin and the mucous membranes	–	–	Skin sloughing covering <10% BSA with associated signs (e.g., erythema, purpura, epidermal detachment, and mucous membrane detachment)	Skin sloughing covering 10–30% BSA with associated signs (e.g., erythema, purpura, epidermal detachment, and mucous membrane detachment)	Death
Toxic epidermal necrolysis	A disorder characterized by greater than 30% total body skin area separation of dermis. The syndrome is thought to be a	–	–	–	Skin sloughing covering ≥30% BSA with associated symptoms (e.g., erythema, purpura, or	Death

	hypersensitivity complex affecting the skin and the mucous membranes				epidermal detachment)	
**Infections and infestations**						
Urinary tract infection	A disorder characterized by an infectious process involving the urinary tract, most commonly the bladder and the urethra	–	Localized; local intervention indicated (e.g., topical antibiotic, antifungal, or antiviral)	IV antibiotic, antifungal, or antiviral intervention indicated; radiologic or operative intervention indicated	Life-threatening consequences; urgent intervention indicated	Death
**Laboratory abnormalities**						
Hemoglobin decreased (anemia)	A disorder characterized by a reduction in the amount of hemoglobin in 100 mL of blood. Signs and symptoms of anemia may include pallor of the skin and mucous membranes, shortness of breath, palpitations of the heart, soft systolic murmurs, lethargy, and fatigability	Hgb <LLN–10.0 g/dL; <LLN–6.2 mmol/L; <LLN–100 g/L	Hgb <10.0–8.0 g/dL; <6.2–4.9 mmol/L; <100–80 g/L	Hgb <8.0 g/dL; <4.9 mmol/L; <80 g/L; transfusion indicated	Life-threatening consequences; urgent intervention indicated	Death
Lymphocyte count decreased	A finding based on laboratory test results that indicate a decrease in number of lymphocytes in a blood specimen	<LLN–800/mm^3; <LLN–0.8 x 10e9/L	<800–500/mm^3; <0.8–0.5 x 10e9/L	<500–200/mm^3; <0.5–0.2 x 10e9/L	<200/mm^3; <0.2 x 10e9/L	–
Neutrophil count decreased	A finding based on laboratory test results that indicate a decrease in number of neutrophils in a blood specimen	<LLN–1500/mm^3; <LLN–1.5 x 10e9/L	<1500–1000/mm^3; <1.5–1.0 x 10e9/L	<1000–500/mm^3; <1.0–0.5 x 10e9/L	<500/mm^3; <0.5 x 10e9/L	–
AST increased	A finding based on laboratory test results that indicate an increase	>ULN–3.0 x ULN	>3.0–5.0 x ULN	>5.0–20.0 x ULN	>20.0 x ULN	–

	in the level of aspartate aminotransferase (AST or SGOT) in a blood specimen					
Creatinine increased	A finding based on laboratory test results that indicate increased levels of creatinine in a biological specimen	>1–1.5 x baseline; >ULN–1.5 x ULN	>1.5–3.0 x baseline; >1.5–3.0 x ULN	>3.0 baseline; >3.0–6.0 x ULN	>6.0 x ULN	–
Sodium decreased (hyponatremia)	A disorder characterized by laboratory test results that indicate a low concentration of sodium in the blood	<LLN–130 mmol/L	–	<130–120 mmol/L	<120 mmol/L; life-threatening consequences	Death
ALT increased	A finding based on laboratory test results that indicate an increase in the level of alanine aminotransferase (ALT or SGPT) in the blood specimen	>ULN–3.0 x ULN	>3.0–5.0 x ULN	>5.0–20.0 x ULN	>20.0 x ULN	–
Lipase increased	A finding based on laboratory test results that indicate an increase in the level of lipase in a biological specimen	>ULN–1.5 x ULN	>1.5–2.0 x ULN	>2.0–5.0 x ULN	>5.0 x ULN	–
Albumin decreased (hypoalbuminemia)	A disorder characterized by laboratory test results that indicate a low concentration of albumin in the blood	<LLN–3 g/dL; <LLN–30 g/L	<3–2 g/dL; <30–20 g/L	<2 g/dL; <20 g/L	Life-threatening consequences; urgent intervention indicated	Death
Phosphate decreased (hypophosphatemia)	A disorder characterized by laboratory test results that indicate a low concentration of phosphates in the blood	<LLN–2.5 mg/dL; <LLN–0.8 mmol/L	<2.5–2.0 mg/dL; <0.8–0.6 mmol/L	<2.0–1.0 mg/dL; <0.6–0.3 mmol/L	<1.0 mg/dL; <0.3 mmol/L; life-threatening consequences	Death

Potassium decreased (hypokalemia)	A disorder characterized by laboratory test results that indicate a low concentration of potassium in the blood	<LLN–3.0 mmol/L	<LLN–3.0 mmol/L; symptomatic; intervention indicated	<3.0–2.5 mmol/L; hospitalization indicated	<2.5 mmol/L; life-threatening consequences	Death
Potassium increased (hyperkalemia)	A disorder characterized by laboratory test results that indicate an elevation in the concentration of potassium in the blood; associated with kidney failure or sometimes with the use of diuretic drugs	>ULN–5.5 mmol/L	>5.5–6.0 mmol/L	>6.0–7.0 mmol/L; hospitalization indicated	>7.0 mmol/L; life-threatening consequences	Death
Calcium increased (hypercalcemia)	A disorder characterized by laboratory test results that indicate an elevation in the concentration of calcium (corrected for albumin) in blood	Corrected serum calcium of >ULN–11.5 mg/dL; >ULN–2.9 mmol/L; Ionized calcium >ULN–1.5 mmol/L	Corrected serum calcium of >11.5–12.5 mg/dL; >2.9–3.1 mmol/L; Ionized calcium >1.5–1.6 mmol/L; symptomatic	Corrected serum calcium of >12.5–13.5 mg/dL; >3.1–3.4 mmol/L; Ionized calcium >1.6–1.8 mmol/L; hospitalization indicated	Corrected serum calcium of >13.5 mg/dL; >3.4 mmol/L; Ionized calcium >1.8 mmol/L; life-threatening consequences	Death

ADL, activities of daily living; AE, adverse event; ALT, alanine transaminase; AST, aspartate aminotransferase; BSA, body surface area; Hgb, hemoglobin; IV, intravenous; ICU, intensive care unit; LLN, lower limit of normal; NCI CTCAE, National Cancer Institute Common Terminology Criteria for Adverse Events; SGOT, serum glutamic-oxaloacetic transaminase; SGPT, serum glutamic-pyruvic transaminase; TPN, total parenteral nutrition; ULN, upper limit of normal.

[*]Instrumental ADL refers to preparing meals, shopping for groceries or clothes, using the telephone, managing money, etc.

[†]Self-care ADL refers to bathing, dressing and undressing, feeding self, using the toilet, taking medications, and not bedridden.

Supplementary Table 3: Recommended dose modifications for EV-associated AEs of clinical interest [1].

AE	Severity	Dose modification
**Skin reactions**	For persistent or recurrent grade 2 skin reactions	Consider withholding until grade ≤1, then resume treatment at the same dose level or dose reduce by one dose level
	Grade 3 skin reactions	Withhold until grade ≤1, then resume treatment at the same dose level or dose reduce by one dose level
	Suspected SJS or TEN	Immediately withhold, consult a specialist to confirm the diagnosis. If not SJS/TEN, see grade 2–4 skin reactions
	Confirmed SJS or TEN; grade 4 or recurrent grade 3 skin reactions	Permanently discontinue
**Hyperglycemia**	Blood glucose >250 mg/dL	Withhold until elevated blood glucose has improved to ≤250 mg/dL, then resume treatment at the same dose level
**Pneumonitis/ILD**	Grade 2	Withhold until grade ≤1, then resume treatment at the same dose level or consider dose reduction by one dose level
	Grade ≥3	Permanently discontinue
**Peripheral neuropathy**	Grade 2	Withhold until grade ≤1, then resume treatment at the same dose level (if first occurrence). For a recurrence, withhold until grade ≤1, then resume treatment reduced by one dose level
	Grade ≥3	Permanently discontinue
**Other nonhematologic toxicity**	Grade 3	Withhold until grade ≤1, then resume treatment at the same dose level or consider dose reduction by one dose level
	Grade 4	Permanently discontinue
**Hematologic toxicity**	Grade 3, or grade 2 thrombocytopenia	Withhold until grade ≤1, then resume treatment at the same dose level or consider dose reduction by one dose level
	Grade 4	Withhold until grade ≤1, then reduce dose by one dose level or discontinue treatment

Grading based on NCI CTCAE Version 4.03 (Supplementary Table 2) [3]. For more information, refer to the latest EV prescribing information.

AE, adverse event; EV, enfortumab vedotin; ILD, interstitial lung disease; NCI CTCAE, National Cancer Institute Common Terminology Criteria for Adverse Events; SJS, Stevens–Johnson syndrome; TEN, toxic epidermal necrolysis.

Supplementary Table 4. Dose reduction schedule for EV [1].

	**Dose level**
Starting dose	1.25 mg/kg up to 125 mg
First dose reduction level	1.0 mg/kg up to 100 mg
Second dose reduction level	0.75 mg/kg up to 75 mg*

EV, enfortumab vedotin.

Patients requiring a dose reduction may be re-escalated by one dose level (i.e., patients reduced to 0.75 mg/kg may only be re-escalated to 1 mg/kg) provided the toxicity does not require study drug discontinuation and has returned to baseline or grade ≤1. If the toxicity recurs, re-escalation will not be permitted. For more information, refer to the latest EV prescribing information.

*In the Dose Escalation Cohort, patients with a starting dose of 1.0 mg/kg were allowed up to a two-dose reduction level down to 0.5 mg/kg during the dose-limiting toxicity assessment period to ensure patient safety while maintaining treatment at a clinically active dose level [4].

Supplementary Table 5: Recommended dose modifications for Pembro-associated AEs of clinical interest [2].

AE	Severity	Dosage modification
**irAEs**		
Pneumonitis	Grade 2	Withhold*
	Grade 3 or 4	Permanently discontinue
Colitis	Grade 2 or 3	Withhold*
	Grade 4	Permanently discontinue
Hepatitis with no tumor involvement of the liver	AST or ALT increases to more than three and up to eight times ULN<br>or<br>Total bilirubin increases to more than 1.5 and up to three times ULN	Withhold*
	AST or ALT increases to more than eight times ULN<br>or<br>Total bilirubin increases to more than three times ULN	Permanently discontinue
Hepatitis with tumor involvement of the liver†	Baseline AST or ALT is more than one and up to three times ULN and increases to more than five and up to 10 times ULN<br>or<br>Baseline AST or ALT is more than three and up to five times ULN and increases to more than eight and up to 10 times ULN	Withhold*
	ALT or AST increases to more than 10 times ULN<br>or<br>Total bilirubin increases to more than three times ULN	Permanently discontinue
Endocrinopathies	Grade 3 or 4	Withhold until clinically stable or permanently discontinue depending on severity
Nephritis with renal dysfunction	Grade 2 or 3 increased blood creatinine	Withhold*
	Grade 4 increased blood creatinine	Permanently discontinue
Exfoliative dermatologic conditions	Suspected SJS, TEN, or DRESS	Withhold*
	Confirmed SJS, TEN, or DRESS	Permanently discontinue
Myocarditis	Grade 2, 3, or 4	Permanently discontinue
Neurological toxicities	Grade 2	Withhold*
	Grade 3 or 4	Permanently discontinue
**Other AEs**		
Infusion-related reactions	Grade 1 or 2	Interrupt or slow the rate of infusion
	Grade 3 or 4	Permanently discontinue

Grading based on NCI CTCAE Version 4.0 (Supplementary Table 2) [3]. For more information, refer to the latest Pembro prescribing information.

AE, adverse event; ALT, alanine aminotransferase; AST, aspartate aminotransferase; DRESS, drug rash with eosinophilia and systemic symptoms; irAE, immune-related adverse event; NCI CTCAE, National Cancer Institute Common Terminology Criteria for Adverse Events; Pembro, pembrolizumab; SJS, Stevens–Johnson syndrome; TEN, toxic epidermal necrolysis; ULN, upper limit of normal.

*Resume in patients with complete or partial resolution (grades 0 to 1) after corticosteroid taper. Permanently discontinue if no complete or partial resolution within 12 weeks of initiating steroids or inability to reduce prednisone to 10 mg per day or less (or equivalent) within 12 weeks of initiating steroids.

†If AST and ALT are less than or equal to ULN at baseline, withhold or permanently discontinue Pembro based on recommendations for hepatitis with no liver involvement.

Supplementary Table 6. Onset, improvement, and resolution for treatment-related AEs of special interest for EV from EV-103 [5].

	Total number of events,* N	Events* with improvement, n (%)	Events* with resolution, n (%)	Median time to:		
				Onset of first event, months (range)	Improvement,† months (range)	Resolution,‡ months (range)
**Skin reaction**	65	2 (3.1)	58 (89.2)	0.7 (0.1–15.7)	0.7 (0.2–1.2)	1.2 (0.1–27.1)
**Peripheral neuropathy**	43	20 (46.5)	10 (23.3)	2.4 (0.7–12.5)	6.6 (0.3–27.4)	7.2 (3.5–19.1)
**Hyperglycemia**	7	1 (14.3)	6 (85.7)	0.5 (0.3–3.5)	0.5 (0.5–0.5)	1.6 (0.5–19.7)

Data reflect patients with urothelial cancer who received at least one dose of EV + Pembro from the EV-103 Dose Escalation Cohort/Cohort A (N = 45). Treatment consisted of EV 1.25 mg/kg (on days 1 and 8 of a 21-day cycle) and Pembro 200 mg (on day 1 of a 21-day cycle).

AE, adverse event; EV, enfortumab vedotin; Pembro, pembrolizumab.

*Patients could have had more than one event.

†Improvement defined as at least one grade improvement from the worst grade at the last assessment.

‡Resolution defined as a return to baseline grade or better at the last assessment or recovered outcome.

Supplementary Table 7. Monitoring for AEs of clinical interest in patients on EV + Pembro.

Patients should be counselled to report any new and/or worsening symptoms that can be associated with EV + Pembro to their care team to determine if prompt intervention is necessary.

	**Potential signs/symptoms**	**Potential screening questions**
**Skin reactions** [6, 7]	Warning signs for SJS/TEN: blistering, fever, mucosal involvement (ocular, oral, genital), itching, skin changes (color, texture), swelling of extremities, limiting activities of daily living	• Have you noticed any new/worsening rashes?<br>• Have you experienced fever or other generalized symptoms?<br>• Have you noticed any blistering in/around your mouth, eyes, and/or genitals?
**Peripheral neuropathy** [8]	Sensory peripheral neuropathy: pain/burning, numbness, tingling, or loss of sensation<br>Motor peripheral neuropathy: loss of coordination, muscle weakness	• Have you noticed sensations such as pain, burning, numbness, or tingling anywhere in your body?<br>• Have you had trouble walking or performing daily tasks?<br>• Ask the patient to pick up a coin, pen, or other object, button or unbutton a shirt. Difficulty with these tasks may indicate onset of peripheral neuropathy
**Hyperglycemia/diabetes mellitus** [6]	Frequent urination, increased thirst, blurred vision, confusion, drowsiness, loss of appetite, fruity breath smell, nausea, harder to control blood sugar, vomiting, or stomach pain	• Have you noticed things like increased urination or thirst?<br>• Have you experienced periods of confusion or drowsiness?
**GI events** [6]	Abdominal pain, nausea, cramping, blood or mucus in stool, changes in bowel habits, fever, abdominal distention, obstipation, constipation	• Have you noticed any changes in your bowel movements from what is normal, such as change in appearance or consistency?<br>• Have you experienced any recent pain in your abdomen? Can you describe the pain and how frequently it occurs?
**Fatigue** [6, 9]	Weight gain, hair loss, cold intolerance, constipation, depression, mood changes, loss of libido, limiting activities of daily living	• Have you noticed any changes in being able to complete normal activities?<br>• Have you experienced periods of low mood or energy?<br>• Have you noticed changes in your weight or appetite?
**Pneumonitis/interstitial lung disease** [10]	Cough, decreased activity tolerance, fever, chest pain, shortness of breath, trouble breathing	• Have you had any coughing or chest pain?<br>• Are you able to do all the activities you normally do without difficulty breathing?
**Ocular disorders** [6]	Dry eye, increased tear production, conjunctivitis, blurred/distorted vision, blind spots, change in color vision, photophobia, tenderness/pain, eyelid swelling, proptosis	• Have you noticed any changes in your vision, including blurred vision or difficulty with lights?<br>• Have you had any pain or dryness in/around your eyes?

AE, adverse event; EV + Pembro, enfortumab vedotin plus pembrolizumab combination; SJS, Stevens–Johnson syndrome; TEN, toxic epidermal necrolysis.

SUPPLEMENTARY FIGURE 1: General management of irAEs related to Pembro [2, 9, 11-14].

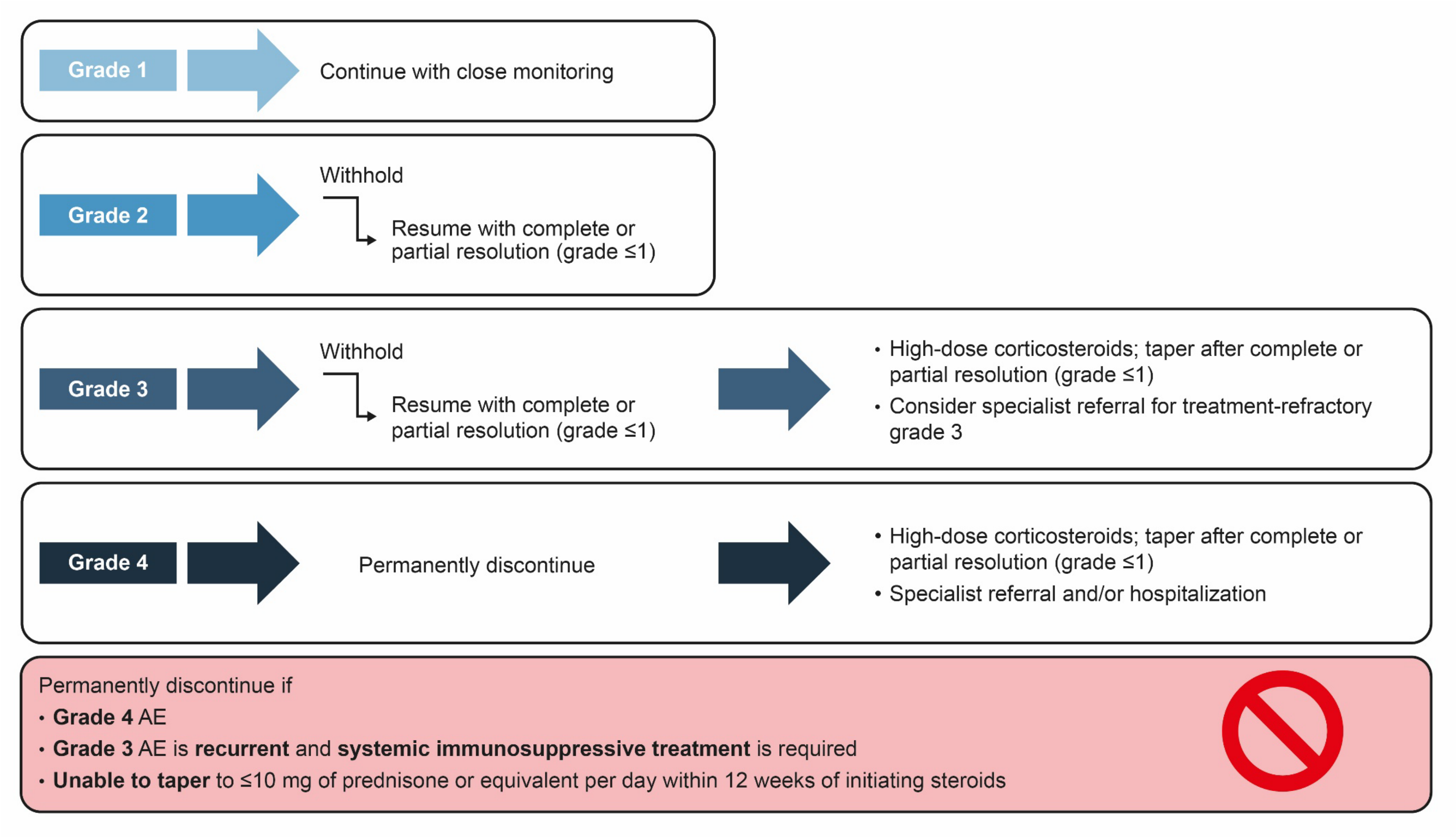

AE, adverse event; irAE, immune-related adverse event; Pembro, pembrolizumab.

References

1. Seagen, Astellas Pharma US Inc.: PADCEV (enfortumab vedotin-ejfv) for injection [prescribing information]. https://astellas.us/docs/PADCEV_label.pdf (2023). Accessed January 18, 2024.
2. Merck & Co.: KEYTRUDA (pembrolizumab) injection [prescribing information]. https://www.merck.com/product/usa/pi_circulars/k/keytruda/keytruda_pi.pdf (2024). Accessed January 17, 2024.
3. National Cancer Institute: Common Terminology Criteria for Adverse Events (CTCAE) Version 4.03. https://ctep.cancer.gov/protocoldevelopment/electronic_applications/docs/CTCAE_4.03.xlsx (2010). Accessed July 21, 2023.
4. Hoimes CJ, Flaig TW, Milowsky MI, Friedlander TW, Bilen MA, Gupta S, et al. Enfortumab vedotin plus pembrolizumab in previously untreated advanced urothelial cancer. J Clin Oncol. 2023;41(1):22-31. doi: 10.1200/JCO.22.01643.
5. Gupta S, Rosenberg J, McKay RR, Flaig TW, Petrylak D, Hoimes CJ, et al. Study EV-103 dose escalation/cohort a: Long-term outcome of enfortumab vedotin + pembrolizumab in first-line (1l) cisplatin-ineligible locally advanced or metastatic urothelial carcinoma (la/mUC) with nearly 4 years of follow-up. Presented at ASCO 2023. 2023
6. National Comprehensive Cancer Network: NCCN Clinical Practice Guidelines in Oncology. Management of Immunotherapy-Related Toxicities, Version 2.2023. https://www.nccn.org/professionals/physician_gls/pdf/immunotherapy.pdf (2023). Accessed May 23, 2023.
7. Lacouture ME, Patel AB, Rosenberg JE, O'Donnell PH. Management of dermatologic events associated with the Nectin-4-directed antibody-drug conjugate enfortumab vedotin. Oncologist. 2022;27(3):e223-e32. doi: 10.1093/oncolo/oyac001.
8. Pace A, Brower B, Conway D, Leis D. Enfortumab vedotin: Nursing perspectives on the management of adverse events in patients with locally advanced or metastatic urothelial carcinoma. Clin J Oncol Nurs. 2021;25(2):E1-E9. doi: 10.1188/21.CJON.E1-E9.
9. Haanen J, Carbonnel F, Robert C, Kerr KM, Peters S, Larkin J, et al. Management of toxicities from immunotherapy: ESMO Clinical Practice Guidelines for diagnosis, treatment and follow-up. Ann Oncol. 2017;28(suppl_4):iv119-iv42. doi: 10.1093/annonc/mdx225.
10. Wang H, Guo X, Zhou J, Li Y, Duan L, Si X, et al. Clinical diagnosis and treatment of immune checkpoint inhibitor-associated pneumonitis. Thorac Cancer. 2020;11(1):191-7. doi: 10.1111/1759-7714.13240.
11. Brahmer JR, Lacchetti C, Schneider BJ, Atkins MB, Brassil KJ, Caterino JM, et al. Management of immune-related adverse events in patients treated with immune checkpoint inhibitor therapy: American society of clinical oncology clinical practice guideline. J Clin Oncol. 2018;36(17):1714-68. doi: 10.1200/JCO.2017.77.6385.
12. Brahmer JR, Abu-Sbeih H, Ascierto PA, Brufsky J, Cappelli LC, Cortazar FB, et al. Society for Immunotherapy of Cancer (SITC) clinical practice guideline on immune checkpoint inhibitor-related adverse events. J Immunother Cancer. 2021;9(6). doi: 10.1136/jitc-2021-002435.
13. Morgado M, Placido A, Morgado S, Roque F. Management of the adverse effects of immune checkpoint inhibitors. Vaccines (Basel). 2020;8(4). doi: 10.3390/vaccines8040575.
14. Kumar V, Chaudhary N, Garg M, Floudas CS, Soni P, Chandra AB. Current diagnosis and management of immune related adverse events (irAEs) induced by immune checkpoint inhibitor therapy. Front Pharmacol. 2017;8:49. doi: 10.3389/fphar.2017.00049.
